# The Influence of Two Distinct Nerves Which Produce Variations in the Color of Venous Blood in Glandular Organs

**Published:** 1859-04

**Authors:** Cl. Bernard


### ARTICLE XXI.

*The Influence of Two Distinct Nerves which produce Variations in the Color of Venous Blood in glandular organs.* By M. Cl. Bernard. Translated from "Archives Générales de Médicine."

In a former communication, M. Bernard had proved that the glandular and muscular venous blood presented an entirely opposite color when the organs were in a state of activity or rest. The learned physiologist has followed up these researches with the design of ascertaining what modifications in composition correspond to these differences of color. But before explaining the chemical view of these phenomena, he proposes to explain the physiological condition of the nervous system which controls these special chemico-organic functions. This is the subject of the present essay, the principal points of which are brought to view.

I. The special chemical conditions which, in glands, cause the venous blood to appear at one time red and at another black, are determined by the influence of two nerves which have distinct origins and possess powers apparently antagonistic to each other.

In other words, there exists a glandular nerve which makes venous blood to flow as red blood, and another nerve which causes the venous blood to become black. Each of these nerves, in order to produce a chemical action on the blood, modifies the mechanical phenomena of the capillary circulation—so that there is established a correllation, both necessary and easy to be understood, between the chemical changes which the blood in the organic tissues undergoes, and mechanical conditions of capillary circulation which are under the immediate influence of the nerves.

The experiments which have led to these results, were made upon the submaxillary gland of the dog, which is peculiarly adapted to this kind of research, on account of the intermittence in its act of secretion, exhibiting very clearly the variations in the color of the venous blood.

The operation is truly a vivisection, the experiment which might be classed among the delicate and laborious operations is much simplified if we take away entirely the digastric muscle. We then have a deep wound, in the bottom of which is found the lower surface of the submaxillary gland also all the vascular and nervous organs, upon which it is easy to experiment.

II. The nerve which causes the venous blood to appear red in the veins of the submaxillary gland, is a small branch which arises from the posterior portion of the lingual branch of the fifth pair; but it is only in contact with the fifth pair, coming really from the seventh, and is principally formed by the corda-tympani. Be this as it may, this glandular nervous filament may be easily reached where it detaches itself from the lingual to be distributed in the submaxillary gland accompanying its excretory duct.

Now, when we observe the submaxillary gland provided with all its nerves and in a state of repose, that is, when there is no discharge from its excretory duct, we observe that its venous blood possesses a very decided dark color, but if now, we bring into exercise the glandular nerve just described, we perceive the venous blood, formerly black, become more and more red, appearing soon thoroughly red as arterial blood, if the nervous irritation had been sufficiently intense. This fact is uniform, and permits the establishment of this physiological proposition, that, whenever the action of the tympanico-lingual nerve is energetic, the venous blood of the submaxillary gland appears red, whilst the blood becomes black whenever this nerve ceases to act or its action ceases to preponderate.

Nothing is easier than to bring experimental proof of this special nervous influence of the tympanico-lingual nerve over the red color of venous blood. When we have exposed the vein and nerve in question, and produced an impression upon the sense of taste, by dropping a little vinegar in the mouth, we see the blood rapidly redden in the vein, because the impression of taste produced upon the tongue, and carried to the nervous centres, has been transmitted by reflex action through the corda-tympani. The proof of the truth of this interpretation of the phenomena is immediately furnished, for, if we cut this nervous filament at the point where it separates from the lingual nerve, we see the venous blood of the gland remains black, and from that moment, notwithstanding the application of vinegar upon the tongue, notwithstanding the impression made upon the sense of taste, the red color of the blood does not appear, because the nervous medium, by which the modifying influence of the blood was conducted, has now been interrupted. But, if now, we irritate, by means of galvanism, the cut extremity of the nerve next to the gland, we see immediately, under the influence of this artificial excitement, the blood in the glandular veins to become red, then resume its black color when this excitement ceases.

This last experiment furnishes a new argument to prove that the red color of the venous blood in the sub-maxillary gland is in proportion to the activity of the tympanico-lingual nerve, and that its black color is in proportion to its physiological inactivity.

In the case of the repose of the gland, the black color which we observe in venous blood is due to a state of activity of another nerve whose power is to render the blood black, and whose permanent influence is antagonistical to the tympanico-lingual nerve, whose action appears to have an intermittent character.

III. The nerve which renders the venous blood in the submaxillary gland, black, is derived from the great sympathetic, and reaches the gland accompanying the artertal branches of the carotid, one, the smaller, penetrating the posterior and superior surface of the gland, the other the principal glandular artery, entering the gland by the side of its excretory duct. These glandular filaments of the sympathetic nervous system, for the most part, arise from the superior cervical ganglion; they anastomose also with filaments coming from other sources, particularly with the mylohyoidian at the point where this nerve crosses the direction of the facial artery.

We have said, that when we observe the submaxillary gland in its physiological state, with its nerves in repose, its venous blood is black; that is, because at this moment, the activity of the great sympathetic, which renders the blood black, exceeds that of the tympanico-lingual, which makes it red. This is easily proved, for if in this state, we cut all the filaments of nerves which go from the great sympathetic to the gland; we see the venous blood lose its black color and take a russet color, which becomes permanent, because the influence of the great sympathetic nerve has been interrupted and does not reach the gland.

But if now we re-establish artificially the activity of this nerve, by applying galvanism to its glandular extremity, we soon see that the venous blood becomes very black, and again resuming its red color as soon as the galvanic influence ceases to act on the nerve. We can then say that the venous blood of the submaxillary gland is black, when the sympathetic nerve acts upon the gland, and that the color is deeper black, in proportion as this nervous influence is energetic.

Thus the variations in the color of the venous blood in the gland are due to two nervous influences well marked and perfectly distinct. How shall we understand the mechanism of this nervous influence over the blood? There is no anatomical continuity, and consequently no direct chemical action possible on the part of the nerves over the globules of the blood, to change their color. It is necessary that there should be other phenomena, intermediate between the nervous action and the chemical change in the globules of the blood; and these intermediate conditions do exist, and consist in the different mechanical changes which each nerve produces in the capillary circulation of the gland.

IV. The mechanical conditions of the capillary circulation, produced in the submaxillary gland by the tympanico-lingual and by the great sympathetic nerves, are exactly the opposites of each other. When the tympanico-lingual nerve is excited, the venous blood appear of a red color and at the same time there comes on a considerable activity in the rapidity of the circulation; in proportion as the venous blood becomes redder, it flows more and more rapidly, and the quantity which flows through the vein is much increased.

In one instance, in which the blood coming from the glandular vein was measured, it was found that during the repose of the gland, when the blood was black, it took sixty-five seconds to collect five cubic centimeters—whilst, when the tympanico-lingual nerve was stimulated by galvanism and the blood flowed of a red color, it required but fifteen seconds to obtain the same quantity of blood, which shows that the circulation in this last instance was four times more rapid than in the former.

When the influence of the great sympathetic predominates, the venous blood is blackened in color, and at the same time its circulation becomes sluggish, the blood flows through the vein with a current, slow in proportion to the intensity of its color, and even if the action of the sympathetic nerve is sufficiently excited, the current of the blood may entirely cease to re-appear, the moment the excitement of the nerve cease, and again is accelerated if the tympanico-lingual nerve is stimulated.

We say then, that the red and black color of the venous blood is in a fixed relation to the activity of the circulation in the submaxillary gland. But this rapidity in the circulation of the blood cannot be produced directly by the nerves, for they have no direct and immediate action on the blood itself. The contraction and expansion of the blood vessels of the gland, can alone explain these modifications in the properties of the blood.

V. It is easy to prove the experiment, that one of the two nerves of the submaxillary gland referred to dilates the vessels, while the other contracts them. The tympanico-lingual nerve enlarges the calibre of the capillary vessels of the gland, and this increase of size is so marked, that when the action of the nerve is intense, the blood passes from the artery into the vein without losing the impulse of the heart, and we then see its exit from the vein of the gland, with an interrupted jet as of a true artery. This venous pulsation disappears as soon as the action of the tympanico-lingual nerve diminishes or entirely ceases.

The sympathetic nerve, on the contrary, contracts and narrows the calibre of the glandular vessels in the most marked manner. When we excite this nerve, the contracted vessels permit less and less blood to pass. The blood, retained in the capillary vessels of the gland, flows feebly with a black color, and exhibits an intensity of color in proportion to the slowness of the current.

Finally, the two nerves which modify the color of the venous blood are two motor nerves, which act primarily by contracting or dilating the blood vessels. The sympathetic is the constrictor nerve of the blood vessels, while the tympanico-lingual is their nerve of dilatation.

VI. In the undisturbed physiological state of the submaxillary gland, we should imagine these two orders of nerves in a state of uniform activity, and antagonistic to each other; so that the efficient nervous force is always due to the influence of that nerve which predominates at that time, and that the special influence of one of these glandular nerves appears not to manifest itself until it has overcome the influence of its opponent. This last phenomenon is very marked, particularly in the case of the tympanico-lingual nerve. If we leave this nerve intact and cut all the filaments of the great sympathetic of the gland, and then put a few drops of vinegar upon the tongue, we see the reddening blood flow through the vein with more intensity, and its pulsations much more energetic than in its natural state, that is, before the sympathetic was divided.

This difference in excitability of the tympanico-lingual nerve is measured in this instance by its proper physiological excitant, the acid substance. This shows the existence, in the submaxillary gland, of a kind of functional libration or balancing, produced by the antagonism of the dilator and constrictor nerve of the capillary blood vessels. The extreme dilatation of the capillary vessels coincides with the direct passage of the red and pulsating blood through the vein. The extreme contraction coincides with a very languid flow of blood, and its black color. Between these extremes, all the intermediate states may be observed.

VII. To recapitulate, the two nerves act only as agents of the dilatation and contraction of the blood vessels. This influence, though not at all differing from that of the motor nerves in general, over the contractile or muscular tissue, nevertheless, by a very natural connection of the phenomena, produces a series of chemico-physical changes in the blood. When the sympathetic nerve, the constrictor of the vessels, is active, the contact between the blood and the glandular elements, is prolonged ; the chemical phenomena, which result from the organic exchanges between the blood and the tissues, has time to take place, and the blood flows of a black color. When, on the contrary, the tympanico-lingual nerve, which dilates the vessels, is active, the passage of the blood through thc gland becomes very rapid, the modifications of the blood which take place in the contact of the blood globules with glandular tissue are accomplished with a different result, and the blood comes from the vein with a very red color, and preserves the appearance of arterial blood.

Thus we may always perceive, between the primitive physiological action of a nerve and the chemical phenomena which result from it, an intermediate action which modifies mechanically the special circulation of the gland.

Thus, through the influence of these two nerves, the submaxillary gland really possesses an individnal circulation, which, in its variations, is independent of the general circulation.

The special nervous system, which thus animates each capillary system and each organic tissue, regulates everywhere the current of blood in its relation to the special physiological acts of the organs. These nervous modifications of the capillary circulation take place in situ, and without the least disturbance to the neighboring organs, still less to the general circulation. Each part is connected with the whole by the common states of the general circulation ; also, by means of the nervous system, each part may have its appropriate circulation and physiology.

Such are the special physiological states of the capillary circulation, produced by the nervous system. There remains now to be ascertained, what is the chemical change in the blood which occurs in these physiological states and gives rise to these alternations of red and black blood in the veins of the gland. This will be the subject of another communication from M. Cl. Bernard.

